# 14-3-3 Proteins in Plant Hormone Signaling: Doing Several Things at Once

**DOI:** 10.3389/fpls.2018.00297

**Published:** 2018-03-13

**Authors:** Lorenzo Camoni, Sabina Visconti, Patrizia Aducci, Mauro Marra

**Affiliations:** Department of Biology, University of Rome Tor Vergata, Rome, Italy

**Keywords:** 14-3-3 proteins, hormone signaling, brassinosteroids, auxin, abscisic acid, gibberellins, ethylene

## Abstract

In this review we highlight the advances achieved in the investigation of the role of 14-3-3 proteins in hormone signaling, biosynthesis, and transport. 14-3-3 proteins are a family of conserved molecules that target a number of protein clients through their ability to recognize well-defined phosphorylated motifs. As a result, they regulate several cellular processes, ranging from metabolism to transport, growth, development, and stress response. High-throughput proteomic data and two-hybrid screen demonstrate that 14-3-3 proteins physically interact with many protein clients involved in the biosynthesis or signaling pathways of the main plant hormones, while increasing functional evidence indicates that 14-3-3-target interactions play pivotal regulatory roles. These advances provide a framework of our understanding of plant hormone action, suggesting that 14-3-3 proteins act as hubs of a cellular web encompassing different signaling pathways, transducing and integrating diverse hormone signals in the regulation of physiological processes.

## Introduction

14-3-3 proteins are highly conserved dimeric proteins with a subunit mass of 30 kDa, widespread in eukaryotic organisms (Aitken et al., 1992; Fu et al., 2000; Huber et al., 2002). They exist in multiple isoforms that form homo- and hetero-dimers (Jones et al., 1995). Among eukaryotes, plants have the largest number of 14-3-3 genes, such as 15 in Arabidopsis, 5 in barley and 8 in rice. In Arabidopsis, the 13 expressed isoforms are designated by Greek letters (χ,ω,ψ,ϕ,υ,λ,ν,κ,μ,ε,o,ι,π) and classified, according to their amino acid sequence similarities, into two distinct groups: the 𝜀 and the non-𝜀 group (Chevalier et al., 2009; Denison et al., 2011).

Although the high degree of sequence conservation among isoforms suggests a corresponding functional redundancy, increasing evidence demonstrates that 14-3-3 isoforms bind to individual targets with different affinities, thereby opening the possibility that regulation of specific processes could be accomplished by single 14-3-3 isoforms (Paul et al., 2012; Pallucca et al., 2014).

Moreover, the large number of isoforms suggests a very high combinatorial complexity in dimer arrangement, which in turn could underlie a fine tuning of their cellular functions.

14-3-3 proteins are, together with the FHA domain-containing proteins, the only phospho-binding regulators identified so far in plants (Chevalier et al., 2009). The common trait of 14-3-3 proteins is their ability to bind target proteins through the recognition of phosphorylated consensus motifs. So far, three 14-3-3 consensus motifs have been proposed: mode I (R/K)XX(pS/pT)XP, mode II (R/K)XXX(pS/pT)XP (Muslin et al., 1996; Yaffe et al., 1997) and the C-terminal mode III (pS/pT)X1-2-COOH (Coblitz et al., 2006; Paiardini et al., 2014), where X is any amino acid and pS/pT represents a phosphoserine or phosphothreonine.

**FIGURE 1:**
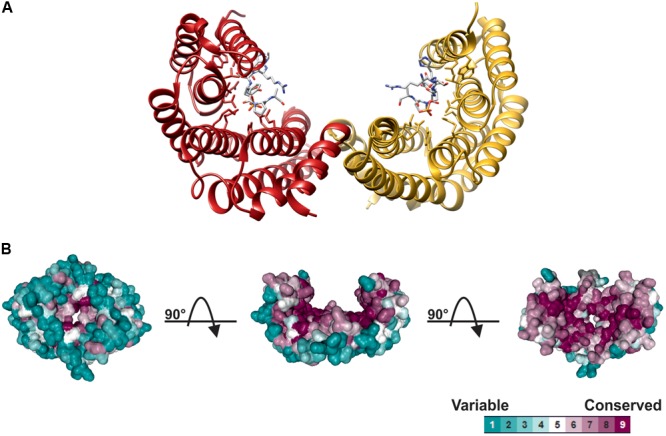
Structure of 14-3-3 proteins. **(A)** Ribbon plot of the human 14-3-3ζ dimer (PDB 1QJB), showing the two monomers (red and yellow ribbon, respectively) complexed with the mode I Raf-1 phosphopeptide (stick models, with carbon atoms colored in gray, oxygen in red, nitrogen in blue and phosphorus in yellow, respectively), which is bound in an extended conformation to the amphipathic groove of each monomer. **(B)** Spacefill structure of 14-3-3ζ shaded according to residue conservation. Structure was analyzed using Consurf (Ashkenazy et al., 2016), aligning 150 14-3-3 isoforms from different eukaryotic organisms. Multiple Sequence Alignment was built using MAFFT (Katoh and Standley, 2013) with residue conservation determined by a maximum likelihood method within Consurf. 14-3-3 structure is shown in three different orientations by sequential 90° rotations, to highlight the high conservation of internal cavity and the variability of the outer surface.

14-3-3 structure and the mechanism of interaction with target proteins has been elucidated upon the determination of X-ray structures in different eukaryotic organisms (Liu et al., 1995; Xiao et al., 1995). As shown in **Figure 1**, monomers consist of nine anti-parallel *α*-helices and associate each other through the *N*-terminal region to assemble the dimeric protein. The 14-3-3 dimer has a characteristic cup-like shape with a highly conserved internal surface and a variable external surface. A conserved amphipathic groove, where the interaction with the phosphorylated target takes place, is present on the concave surface of each monomer, thus implicating that a 14-3-3 dimer can potentially bind two targets at the same time (Yaffe et al., 1997; Ottmann et al., 2007; Taoka et al., 2011).

**FIGURE 2:**
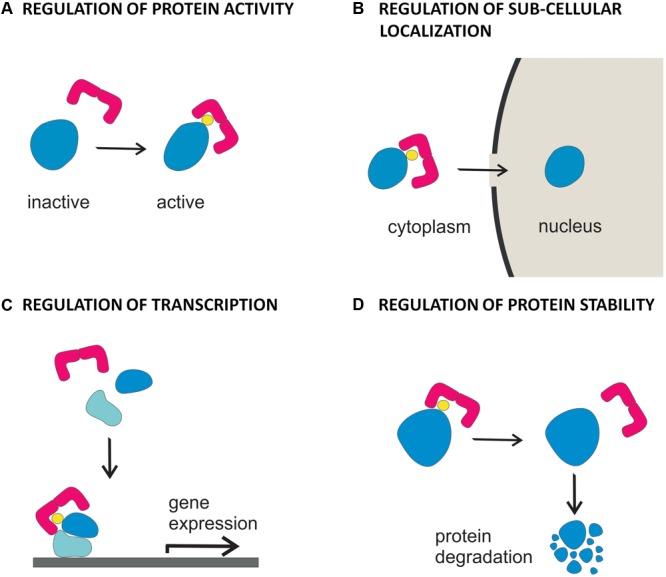
Functional diversity of 14-3-3 proteins in hormone signaling. Binding of 14-3-3 proteins to targets involved in hormone action can result in **(A)** regulation of the activity of the protein, as demonstrated for BKI1 in the BR signaling pathway, for H^+^-ATPase, in IAA and ABA signaling pathways and KAT1, in the IAA signaling pathway **(B)** regulation of the cytoplasmic vs nuclear localization of the protein, as demonstrated for BES1 and BZR1 in the BR signaling pathway and KAT1 in the IAA signaling pathway **(C)** regulation of the assembly and/or the activity of transcriptional elements, as demonstrated for EMBP1/VP1, ABF 1-2 and HvABI5 in the ABA signaling pathway **(D)** control of the turnover of the protein, as demonstrated for ACC synthase and ETO/ETO1-like proteins in the ethylene biosynthesis and signaling pathways.

Depending on the biochemical feature of the phosphorylated target, association of 14-3-3 proteins can have different functional consequences, leading to regulation of its enzymatic activity, subcellular localization, protein stability or alteration of protein-protein interactions (**Figure 2**; Hermeking, 2003; Wilson et al., 2016). In plants, 14-3-3 proteins have been originally identified as component of DNA-protein complexes (Lu et al., 1992) and as co-receptors of the fungal phytotoxin fusicoccin (Korthout and de Boer, 1994; Marra et al., 1994; Oecking et al., 1994). Thereafter, they were found to regulate the plasma membrane H^+^-ATPase (Jahn et al., 1997; Baunsgaard et al., 1998; Fullone et al., 1998) and enzymes of carbon and nitrogen metabolism (Bachmann et al., 1996a,b; Douglas et al., 1997; Toroser et al., 1998; Huber et al., 2002).

Nowadays, a wide range of 14-3-3 clients with a pivotal role in various physiological processes, including growth and development and response to stress has been identified (Moorhead et al., 1999; Aducci et al., 2002; Chevalier et al., 2009; Denison et al., 2011; Jaspert et al., 2011).

In the last years, a growing body of evidence has emerged regarding the involvement of 14-3-3 proteins as key players of different aspects of plant hormone physiology. In this review, we highlight novel insights into the role of 14-3-3 proteins in the regulation of hormonal signaling, biosynthesis and transport.

## Brassinosteroids

Brassinosteroids (BRs) are steroid hormones regulating fundamental functions in plant growth and development, including cell division and elongation, vascular differentiation, flowering, photomorphogenesis, senescence, and responses to environmental stresses. (Clouse and Sasse, 1998).

Genetic and molecular studies in Arabidopsis have greatly advanced the understanding of the BR mode of action, and revealed that 14-3-3 proteins play a complex role in BR signaling by interacting with different members of the BR transduction machinery. Proteomics and yeast two-hybrid screen studies identified the BR receptor Brassinosteroid-Insensitive1 (BRI1), the BRI1 Kinase Inhibitor (BKI1), the BRI1 Suppressor phosphatase (BSU1), the transcription factors BRI1-EMS-Suppressor1 (BES1) and Brassinazole-Resistant1 (BZR1) as 14-3-3 client proteins (Milne et al., 2002; Schoonheim et al., 2007b; Chang et al., 2009).

The first evidence that demonstrated a functional role of 14-3-3 proteins in BR signaling concerned their interaction with BES1/BZR1, which regulate the expression of BR-responsive genes. In the absence of BR, binding of 14-3-3 proteins to phosphorylated BZR1 and BES1 results in their cytoplasmic sequestration and in BR-signaling inhibition. Binding of 14-3-3 proteins occurs upon phosphorylation of Ser^173^ whithin the mode II-type motif RISNpSCP. Accordingly, mutations in Ser^173^ suppress the dwarf phenotype of the receptor mutant (Gampala et al., 2007; Ryu et al., 2007, 2010).

More recently, the interaction with the negative regulator of BR signaling BKI1 has been unveiled (Wang et al., 2011). In the presence of BR, BKI1 is phosphorylated by the BR receptor BRI1 in its C-terminal domain and released into the cytosol, where it associates and antagonizes 14-3-3 proteins, thus promoting BZR1/BES1 translocation into the nucleus. Intriguingly, the interaction with 14-3-3 proteins occurs via an uncommon 14-3-3 mode II motif (RGELFpS^270^APApS^274^), which is also involved in the association with BRI1.

Remarkably, these data suggest that 14-3-3 proteins can act both as negative and positive regulators of the pathway, depending on the BR levels. At low BR levels, they function as negative regulators, whereas at higher BR levels BKI1 and 14-3-3 are released from BRI1 and BES1/BZR1, bind and inhibit each other, thereby allowing the full activation of the BR signaling pathway.

## Auxin

Auxin (indole-3-acetic acid, IAA) is a key regulator of nearly every aspect of plant growth and development, including embryogenesis, lateral root development, vascular tissue differentiation, apical dominance, flower development and tropisms (Teale et al., 2006; Weijers and Wagner, 2016). At the cellular level, IAA controls cell expansion by stimulating the H^+^-ATPase dependent proton extrusion into the cell wall (Rayle and Cleland, 1992). Enzyme activation involves the binding of 14-3-3 proteins to a conserved Thr residue (Thr^947^ in the Arabidopsis AHA1 isoform) at the extreme C terminal end of the autoinhibitory domain of the enzyme, which brings about its displacement, thereby releasing enzyme activity (Fuglsang et al., 1999; Svennelid et al., 1999; Camoni et al., 2000). It was proposed that IAA activates H^+^-ATPase gene transcription (Hager et al., 1991). Successively, it was demonstrated that IAA activates the H^+^-ATPase by a post-translational mechanism. IAA promotes Thr^947^ phosphorylation and subsequent 14-3-3 binding to the Tyr-pThr-Val mode III motif (Takahashi et al., 2012). Although it is not clear whether IAA can activate protein kinases responsible of Thr^947^ phosphorylation, it has recently been demonstrated that IAA action involves the inhibition of a PP2C-D subfamily of type 2C protein phosphatases, that negatively regulates H^+^-ATPase activity (Spartz et al., 2014). In fact, IAA induces the transcription of Small Auxin Up RNA19 (SAUR19), a protein encoded by the *SAUR19-24* subfamily of auxin-induced genes. SAUR19 interacts and inhibits PP2C-D phosphatases, thus promoting H^+^-ATPase phosphorylation and activation. Accordingly, constitutive AtSAUR19 overexpression promotes hypocotyl elongation in tomato plants by a mechanism involving PP2C-D inhibition (Spartz et al., 2014).

Sustained H^+^-ATPase-mediated proton extrusion and growth is dependent on K^+^ influx mediated by inward-rectifier K^+^ channels, required to depolarize the negative potential that thermodynamically inhibits the proton pump. Interestingly, 14-3-3 proteins are also involved in K^+^ channel post-translational regulation. In fact, 14-3-3 proteins bind to and activate the inward-rectifier K^+^ channel1 in *Arabidopsis thaliana* 1 (KAT1) by modifying its open probability (Sottocornola et al., 2006) and by increasing the number of channel delivered at the plasma membrane (Sottocornola et al., 2008). Binding occurs at the mode III motif HLYFSpS^676^N (Saponaro et al., 2017). The overall data suggest that 14-3-3 proteins may function as pivotal regulators of ion transport, integrating different stimuli in the generation and maintenance of the plasma membrane potential.

Localized IAA concentration gradients are essential in different aspects of plant physiology, including tropisms and organ formation. Pin-formed (PIN) proteins, which can be relocated in the cell by endocytic recycling, are a family of IAA transporters essential in the generation of IAA gradients (Naramoto, 2017). It has been recently shown that RNA-interference repression of 𝜀 members of 14-3-3 protein family in Arabidopsis seedlings caused altered polar distribution of IAA and produced related IAA-transport phenotypes (Keicher et al., 2017). These data, despite lack of information concerning molecular 14-3-3 interactors involved in PIN repositioning, clearly point to a fundamental role of the 𝜀 group of 14-3-3 proteins in the regulation of PIN distribution, and IAA transport.

On the whole, accumulated evidence indicates that 14-3-3 proteins are versatile regulators of IAA action, intervening at very different points of the IAA regulatory network: In fact, 14-3-3 proteins can function both downstream, as final transducer of IAA growth-promoting signaling as well as upstream, as wardens of hormone traffic, controlling the formation of IAA gradients.

## Abscisic Acid

Abscisic acid (ABA) is involved in the regulation of key processes of plant development, such as embryogenesis, seed maturation, dormancy, and germination. At the same time, it mediates the response to environmental stresses, including salinity, cold, and drought (Zeevaart and Creelman, 1988).

Knowledge about pathways of ABA signaling has for a long time been fragmentary, until recent studies have shed light on the molecular functions of genetically identified components, including receptors, protein kinases/phosphatases, and different ABA-Responsive Element Binding Factors (ABFs), so that a core model of ABA signaling can be envisaged. In Arabidopsis, ABA is perceived by the Pyrabactin Resistance1 (PYR1)/PYR1-Like (PYL) multigenic family of receptors (Miyakawa et al., 2013). Upon hormone binding, they undergo a conformational change that allow them to associate and inhibit members of clade A of type 2C protein phosphatases (PP2Cs), negative regulators of ABA signaling. PP2C inhibition in turn allows Sucrose non-fermenting-Related kinase2 (SnRK2) kinase activation and phosphorylation of different ABFs, thereby inducing the response (Melcher et al., 2010; Miyakawa et al., 2013). *In vitro* evidence indicates that the SnRK2-type kinase Open Stomata1 (OST1) in Arabidopsis guard cells phosphorylates Thr^451^ of ABF3 within the 14-3-3 binding motif RXX(S/T)XP, conserved in ABFs, thus promoting 14-3-3 association (Sirichandra et al., 2010). Notably, ABA treatment induces ABF3 phosphorylation *in planta* and indirect evidence suggests that Thr^451^ phosphorylation is correlated to enhanced ABF3 stability (Sirichandra et al., 2010).

In Arabidopsis stomata cells and hypocotyls, ABA inhibits the plasma membrane H^+^-ATPase by inducing its dephosphorylation and 14-3-3 release (Hayashi et al., 2011, 2014), while in barley embryonic roots ABA inhibits 14-3-3-activated inward K^+^ channels (van den Wijngaard et al., 2005).

14-3-3 proteins also play a key role in ABA regulated transcription. In fact, they were found as part of transcriptional complexes of ABA-regulated genes. In rice embryogenic cultures and maize embryos, 14-3-3 proteins are part of the complex between the basic leucine zipper (bZIP) transcription factor EmBP1 and Viviparus1 (VP1), which binds to the ABA responsive element Em1a (Schultz et al., 1998), while in Arabidopsis embryos they are associated to ABI3 regulated AtEm1 promoter (del Viso et al., 2007). Moreover, in embryonic barley roots 14-3-3 proteins have a function in the ABA regulated transcriptional cascade. In fact, RNAi-mediated silencing of individual 14-3-3 isoforms resulted in reduction of the expression of a reporter gene controlled by the ABA-inducible promoter ABA-Response Complex3 (ABRC3). Yeast two-hybrid screen allowed to identify the seed specific ABI1-3 and ABI5 proteins, belonging to the ABF family of bZip transcription factors, as 14-3-3 interactors (Schoonheim et al., 2007a, 2009). Interestingly, in this system ABA also increases the expression of four out of five 14-3-3 barley genes, thus revealing a reciprocal relationship between ABA and 14-3-3 proteins: they act as signaling effectors and in turn are under transcriptional control by ABA, according to a positive feedback circuit.

## Gibberellins

Gibberellins (GAs) are a wide family of tetracyclic diterpenoid molecules that regulates fundamental plant processes, like germination and stem elongation, besides many other aspects of plant growth and development, such as floral initiation, pollen development, leaf expansion, trichome and anther development (Richards et al., 2001). Initial research demonstrated that 14-3-3 proteins are involved in the control of the GA biosynthetic pathway. GAs regulate their own biosynthesis by a negative feedback mechanism involving the bZip transcriptional activator Repression of Shoot Growth (RSG), which binds to the promoter of the biosynthetic enzyme *ent* kaurene oxidase (GA3) gene (Fukazawa et al., 2000). In transgenic tobacco plants, 14-3-3 proteins were co-precipitated with RSG. Mutation of Ser^114^ whithin the sequence RSLpSVD impaired 14-3-3 binding, inducing RSG translocation into the nucleus and increased transcription (Igarashi et al., 2001). Moreover, RSG translocation into the nucleus was promoted by a reduction of GA levels (Ishida et al., 2004). These lines of evidence clearly indicate that 14-3-3 proteins participate to GA biosynthesis as negative regulators, sequestering in the cytoplasm the transcriptional regulator RSG. 14-3-3 binding is mediated by RSG phosphorylation promoted by *Nicotiana tabacum* Calcium-Dependent Protein Kinase (NtCDPK1), which also functions as a scaffold protein, bridging 14-3-3 proteins to RSG (Ito et al., 2014).

More recent work demonstrates that 14-3-3 proteins are also involved in GA signaling. In barley aleurone cells, isoform-specific 14-3-3 RNAi-mediated silencing inhibits GA activation of a reporter gene under the control of the α-amylase promoter. In this system, a possible role for 14-3-3 proteins in the coordination of GA and ABA signaling has emerged. In fact, the overexpression of ABA responsive, 14-3-3-interacting transcription factors ABF1-3 impairs GA action, indicating that they act as negative regulators of GA signaling and that 14-3-3 proteins may function by sequestering ABF1-3 in the cytoplasm. However, the mechanism of 14-3-3 action is still unclear, since abolition of 14-3-3/ABF1-2 interaction affects their ABA-dependent transactivation activity, while the same deletion does not influence their inhibitory activity in GA signaling (Schoonheim et al., 2009).

## Ethylene

The gaseous hormone ethylene influences several aspects of plant growth and development, including germination, cell expansion, leaf and flower senescence and abscission, fruit ripening, resistance to pathogen infection and adaptation to stress conditions (Bleecker and Kende, 2000). Ethylene is synthesized from the amino acid methionine. Conversion of S-adenosyl-methionine (SAM) in 1-aminocyclopropane-1-carboxylic acid (ACC), catalyzed by a family of ACC synthase enzymes (ACS), is the rate-limiting step of ethylene synthesis (Wang et al., 2002). In the last years, the role of 14-3-3 proteins in the post-translational regulation of ethylene biosynthesis has emerged. In fact, it has been demonstrated the ability of 14-3-3 proteins to interact *in vivo* with different ACS isoforms (Chang et al., 2009; Huang et al., 2013; Yoon and Kieber, 2013; Catalá et al., 2014). 14-3-3 proteins likely bind to ACS through non-canonical binding sites as neither the mode I nor mode II binding sites are present in ACS proteins. Interestingly, 14-3-3 proteins interact also with components involved in the regulation of ACS stability, the Ethylene-Over-producer1 (ETO1)/ETO1-Like (EOLs) proteins. They are part of a Cullin-3 E3 ubiquitin ligase complex that targets ACS protein for 26S-proteasome-mediated degradation. Binding of 14-3-3 proteins destabilizes ETO1/EOLs, thereby blocking the Cullin-3 E3 ubiquitin ligase activity and consequently the proteasome-mediated ACS degradation (Yoon and Kieber, 2013).

However, contrasting results have been obtained studying the Arabidopsis mechanism which regulates freezing tolerance and cold acclimation. The 14-3-3 ψ isoform, encoded by the *RARE COLD INDUCIBLE 1A* (*RCA1*) gene, interacts with ACS, negatively regulating its stability and consequently lowering ethylene production (Catalá et al., 2014).

## Cytokinins and Other Hormones

In the last years, data on the interaction of 14-3-3 proteins with components of signaling pathways of other hormones have been reported. Although the physiological relevance of these interactions is still to be ascertained, these data allow to envisage a regulatory role of 14-3-3 proteins in the action of cytokinin, jasmonate (JA) and salicylic acid (SA).

Affinity chromatography/mass spectrometry and yeast two hybrid screen (Schoonheim et al., 2007b; Chang et al., 2009; Jaspert et al., 2011) in barley and Arabidopsis allowed to identify different enzymes of cytokinin metabolism (CKX3, cytokinin oxidase) and signaling components (Arabidopsis Response Regulators ARR2 and ARR12 and Cytokinin Response Factor CRF6) as 14-3-3 interacting proteins.

Immunoprecipitation and Surface Plasmon Resonance (SPR) experiments demonstrated that 13-lipoxygenase (13-LOX) interacts with 14-3-3 proteins in barley embryos. This enzyme controls lipid metabolism, which is a key process not only in germination, but also in the biosynthesis of the stress responsive hormone JA (Holtman et al., 2000a,b). Furthermore, the 14-3-3 λ isoform was identified by a yeast two-hybrid screen as an interactor of the RPW8.2 gene product, a R receptor that mediates SA-dependent resistance to the biotrophic fungal pathogens *Golovinomyces* spp. Accordingly, overexpression of *GF14λ* gene enhanced, whereas downregulation hampered, the SA-dependent resistance (Yang et al., 2009).

## Concluding Remarks

This review highlighted the involvement of 14-3-3 proteins in plant hormone regulation, an emerging topic concerning 14-3-3 functions. In fact, whereas a direct regulatory role of 14-3-3 proteins in diverse aspects of plant physiology, from primary metabolism to ion transport, has been well documented, a growing body of evidence indicates that 14-3-3 participate also to a secondary level of regulation, i.e., by affecting hormone signal transduction pathways and biosynthesis. The emerging picture is complex, reflecting the high number of targets and the multiplicity of the 14-3-3 effects. In fact, their distinctive trait to bind to phosphorylated targets ensures that 14-3-3 proteins interact simultaneously with multiple components and/or at different steps of hormone signaling networks, implicating that they can carry out diverse and even opposing functions in different pathways (e.g., GA, ABA), or in the same pathway (e.g., BR). Furthermore, additional complexity may arise from reciprocal regulation by hormones of 14-3-3 concentrations, as well as from specificity/redundancy of functions of the numerous plant 14-3-3 isoforms, for which information is still scarce. However, even though a unique rationale of 14-3-3 mode of action in hormone regulation cannot be envisaged, some common traits can be inferred from so far available data.

A recurring regulatory mechanism of 14-3-3 action is exerted at the transcriptional level, that 14-3-3 proteins influence by functioning as adaptor proteins (e.g., ABA) or altering the sub-cellular localization (e.g., GA, BR) or stability (e.g., ABA) of diverse families of transcriptional regulators. Intriguingly, recent work suggests that the control of stability or localization of transcription factors, shared by different pathways, may represent a mechanism which allows 14-3-3 proteins to integrate multiple hormone pathways, thus controlling a specific physiological process (e.g., ABFs factors in GA and ABA signaling). Alternatively, 14-3-3 can regulate hormone action at the post-translational level, by modulating the activity of client proteins in signaling cascades (e.g., IAA, ABA) or in biosynthetic pathways (e.g., ethylene, GA). Simultaneous control of multiple 14-3-3 clients (e.g., H^+^-ATPase, KAT1) within the same pathway (e.g., IAA) or regulation of targets (e.g., H^+^-ATPase) participating to different pathways (e.g., IAA, ABA) provides a way for 14-3-3 proteins to coordinate the action of diverse hormones in the control of a specific physiological process.

Data reported in this review depict a complex scenario, where a network of interactions among 14-3-3 proteins and their targets finely regulate hormone signaling and homeostasis. It is conceivable that in the next future the identification of novel clients will increase the complexity of the 14-3-3 signaling web. Hence, in order to get a deeper insight, future work should be addressed toward a detailed biochemical characterization of interactions, including the identification of the binding sequence as well as the functional results, i.e., whether it involves modification of protein activity, sub-cellular localization or stability (**Figure 2**). Moreover, to solve the 14-3-3 specificity vs. redundancy dilemma, it will be crucial to get information about the relative affinities of different isoforms toward each single 14-3-3 client.

## Author Contributions

LC and MM wrote the manuscript. SV and PA contributed to the writing of manuscript and critically revised it for essential intellectual content. LC prepared the figures.

## Conflict of Interest Statement

The authors declare that the research was conducted in the absence of any commercial or financial relationships that could be construed as a potential conflict of interest.
